# Lost in Learning: Hypertext Navigational Efficiency Measures Are Valid for Predicting Learning in Virtual Reality Educational Games

**DOI:** 10.3389/fpsyg.2020.578154

**Published:** 2020-11-25

**Authors:** Chris Ferguson, Herre van Oostendorp

**Affiliations:** Department of Information and Computing Sciences, Utrecht University, Utrecht, Netherlands

**Keywords:** VR game, game analytics, lostness measures, predictive validity, learning

## Abstract

The lostness measure, an implicit and unobtrusive measure originally designed for assessing the usability of hypertext systems, could be useful in Virtual Reality (VR) games where players need to find information to complete a task. VR locomotion systems with node-based movement mimic actions for exploration and browsing found in hypertext systems. For that reason, hypertext usability measures, such as “lostness” can be used to identify how disoriented a player is when completing tasks in an educational game by examining steps made by the player. An evaluation of two different lostness measures, global and local lostness, based on two different types of tasks, is described in a VR educational game using 13 college students between 14 and 18 years old in a first study and extended using 12 extra participants in a second study. Multiple Linear Regression analyses showed, in both studies, that local lostness, and not global lostness, had a significant effect on a post-game knowledge test. Therefore, we argued that local lostness was able to predict how well-participants would perform on a post-game knowledge test indicating how well they learned from the game. In-game experience aspects (engagement, cognitive interest, and presence) were also evaluated and, interestingly, it was also found that participants learned less when they felt more present in the game. We believe these two measures relate to cognitive overload, which is known to have an adverse effect on learning. Further research should investigate the lostness measure for use in an online adaptive game system and design the game system in such a way that the risk of cognitive overload is minimized when learning, resulting in higher retention of information.

## 1. Introduction

Within education, serious games are often used as learning tools. A key aim of many of these educational serious games is information acquisition, without explicit teaching, where items must be discovered to proceed further in the game. In particular, narrative-centered discovery games have considerable learning potential. In these games, players are transported to another place and time period and must explore the environment, completing tasks to discover items that reveal the in-game story (Malone and Lepper, 1987; Lester et al., 2014). This stimulates players to construct appropriate mental models (Wasserman and Banks, 2017; Furlough and Gillan, 2018).

When it comes to these types of educational games, in-game analytics is a major focus as it is beneficial to know how a player is performing during play. For example, these analytics can help to characterize players when carrying out such information-searching tasks (e.g., as chaotic or goal-oriented) and can be instrumental in improving the game (e.g., where confusion arises). Most importantly, the analytics can be used to dynamically improve (adapt) the game based on the in-game performance of the player, which may lead to better learning (Van Oostendorp et al., 2014).

There are many different in-game analytics and analytic systems (see e.g., Drachen and Schubert, 2013; Westera et al., 2014; Loh et al., 2015). However, game analytics are often based on shallow interaction data (Perez-Colado et al., 2018) and their success in predicting learning can be limited or unknown. Often, data mining algorithms are used rather than a full analysis of the task that needs to be performed, along with the cognitive processes and cognitive problems that players encounter whilst carrying out these tasks. Consequently, this leads to a multitude of data being collected to develop analytics that are not aligned with the mental operations a player needs to carry out in building an adequate mental model to effectively complete a task (Furlough and Gillan, 2018). In contrast, we aim to sample characteristic difficulties in executing and orchestrating specific information processes needed to solve an (information search) task (Elshout, 1976). With this in mind, in this study game analytics will be developed based on a cognitive analysis of the search task.

Due to the perceived high engagement and assumed higher knowledge transfer of VR, there has been a transition from traditional PCs to VR serious games on different domains. These include evacuation training and hazard awareness (Feng et al., 2018). This is of particular interest as these two domains make use of spatial activities, particularly navigation, in the case of evacuation training. In a parallel study (Ferguson et al., 2020b) a VR educational game condition was compared to a traditional, non-VR desktop condition. The research question was whether or not VR had a positive effect on the retention of educational content and navigational efficiency. We define navigational efficiency as how efficiently a user completes a goal (i.e., finding information), in terms of taking the shortest path by visiting the minimum number of locations without revisiting the same locations multiple times (Smith, 1996). In this study, VR was found to have a significant positive benefit on spatial activities, in the form of increased retention of spatial knowledge, and on navigational efficiency as participants were better able to efficiently navigate the virtual environment. As these VR games for learning become more prevalent, analytics should be investigated for VR educational games that may be more appropriate than analytics already available and therefore we will examine whether we can use navigational efficiency as an implicit indicator of learning performance. Because we have to focus on the performance of players in the VR condition, we do not want to confound the VR data from that parallel study with non-VR data in correlational analyses, and we restrict ourselves to only using the VR data. Therefore, this paper will focus on only the VR participants from that parallel study (Ferguson et al., 2020b) to investigate if possible implicit (unobtrusive) navigational efficiency measures are valid for predicting learning in certain types of VR educational games.

We present a study focused on the implicit and unobtrusive measuring of the navigation patterns of players during playing a Virtual Reality (VR) educational game. We assume that all interactions a player makes in a game are related to the learning process, and, thus, can form a useful and valid indicator of the learning process and, consequently, predict the final learning results. Our assumption is, therefore, that game analytics relevant to learning should be based on information processing characteristics indicative of learning, and thus correlate with learning. The predictive validity of these so-called cognition-based game analytics concerning learning will be examined in this study, at the same time considering other potentially relevant factors, such as game experience aspects, that is, how a user feels whilst playing the game.

## 2. Theoretical Background

### 2.1. Navigation and the Issue of Disorientation

As mentioned above, many educational games require navigating around a virtual environment to complete tasks to acquire information as part of the learning process. Unfortunately, navigating through such games can be challenging and lead to disorientation, where players lose their sense of location and direction (Conklin, 1987; Head et al., 2000). This happens when navigation is too much of a cognitive burden and leads to cognitive overload (Gwizdka and Spence, 2007), i.e., an excessive amount of load placed on a person's working memory when carrying out a task (Chen, 2016).

Disorientation and cognitive overload when navigating large, complex systems is a well-known problem within hypertext systems like websites (Conklin, 1987; Edwards and Hardman, 1989; Gwizdka and Spence, 2007). Edwards and Hardman (1989) coined the term “Lost in Hyperspace” for this phenomenon. It is a key issue as cognitive overload leads to inhibited learning (Sweller et al., 2011) when a user simultaneously has to spend limited cognitive resources to both navigation and comprehension (Sharples, 1999; Bolter, 2000). Navigation is part of the learning process and navigation problems leading to disorientation have a negative effect on learning (Sweller et al., 2011). Disorientation can be measured by navigational efficiency, where a player who is inefficient during navigation (low navigational efficiency) can be classified as disoriented and an efficient player (with high navigational efficiency) can be classified as not disoriented at all. Therefore, to provide analytics for learning, a metric must be identified that can measure the navigational efficiency of a player also within a VR game. We argue that navigation in VR systems with node-based movements can be compared with navigation as occurring in hypertext systems, and, for that reason, a navigation efficiency measure with validated success in hypertext environments can also be useful in a VR environment

**Figure 1:**
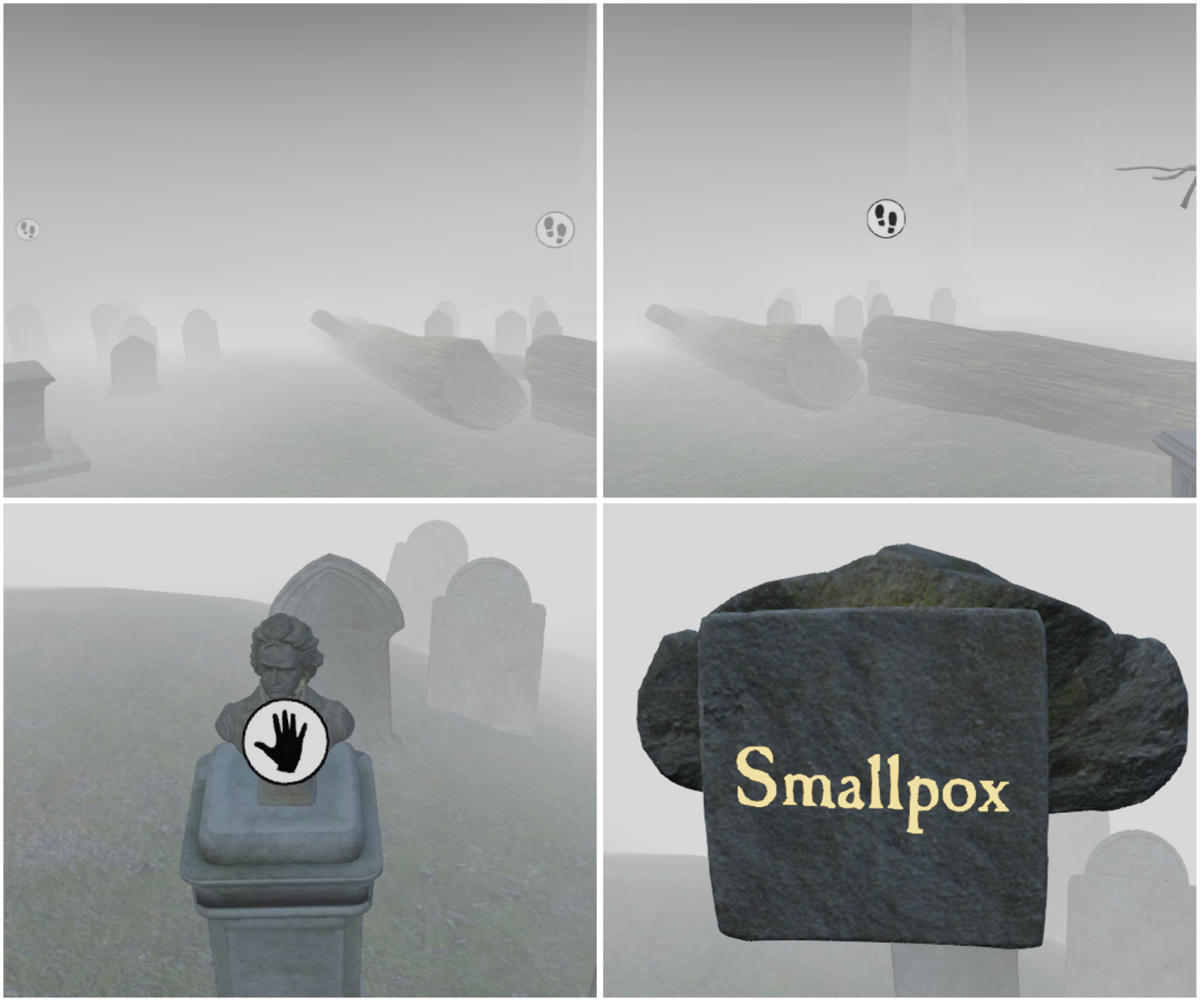
The node-based movement system within “*the Chantry”*. Players can only move to locations shown by a footsteps icon and pick up items when shown a hand icon. Some objects have a label underneath, or on the back, representing a task.

**Figure 2:**
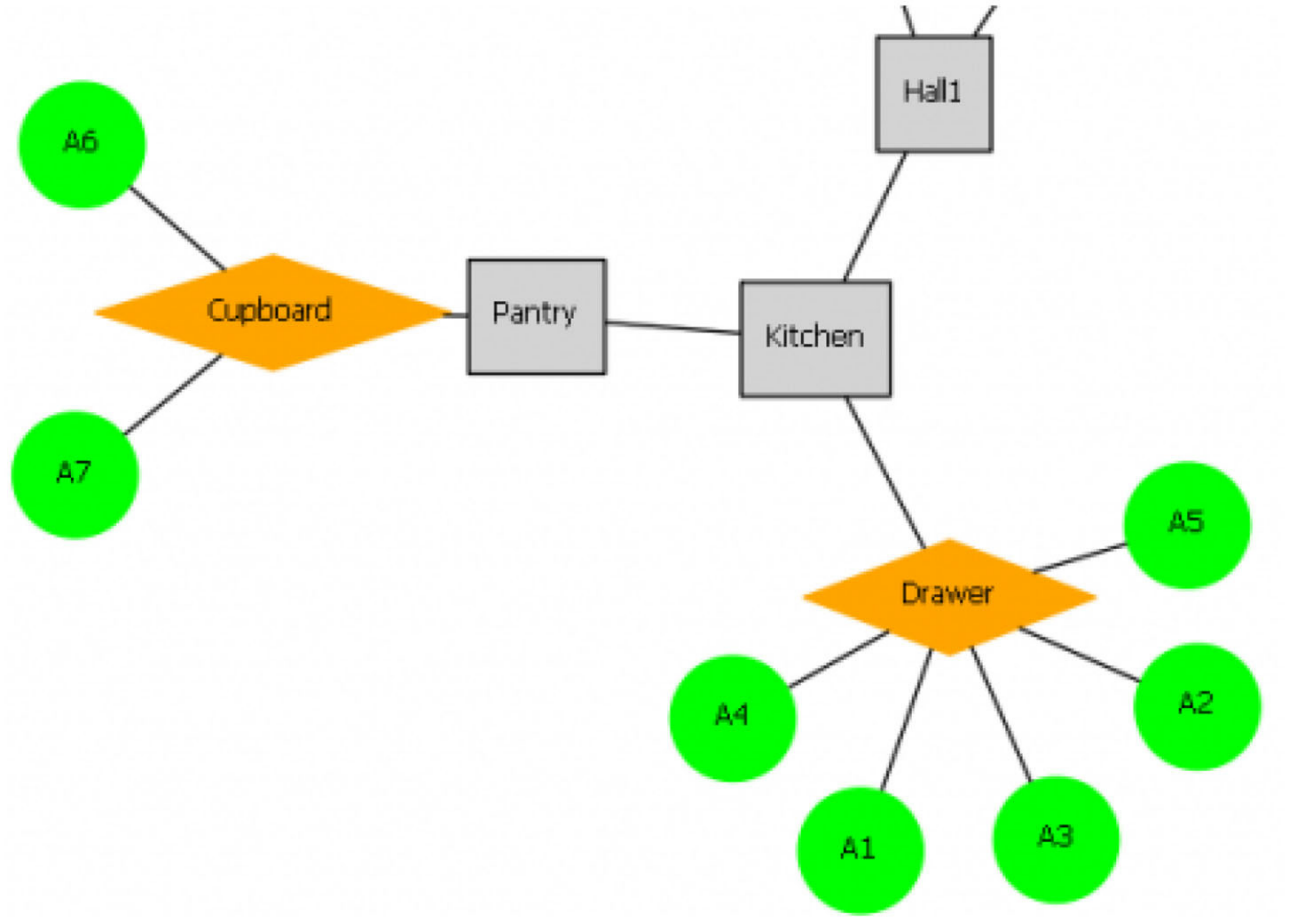
An example of the spatial graph used in “*the Chantry”*. This represents the locations and objects that a player can access [fig:spatial]. Gray nodes represent overall locations (rooms) that a player can visit, orange nodes represent items a player can interact with to reveal objects, and green nodes represent objects that can be picked up and interacted with.

Node-based movement systems with fast movement speeds are used in VR due to its effectiveness in reducing the likelihood of motion sickness (Habgood et al., 2018). This system of movement has the additional effect of restricting players to only visiting set locations, allowing developers to guide players, assisting them with navigation, rather than them being able to roam freely around the environment as in traditional first-person games (see Figure 1). When using this kind of movement systems, the locations a player can visit are stored in a spatial node graph, a link-node model where nodes are shown as being linked together (see Figure 2). A player can move directly to another node only if the target node and the current node are linked. Otherwise, they must navigate through other nodes, following a path to the target node similar to navigating a hypertext system.

User navigation behavior in the context of web information search tasks consists of sequentially moving through a set of pages, deciding at each step (each hyperlink) where to go. It can be divided into two main stages of cognitive processing: attention cycle and action-selection cycle (Kitajima et al., 2000; Karanam et al., 2016; Van Oostendorp et al., 2017). The attention cycle is further divided into two stages: parsing the web page in high-level schematic regions and focusing on one of these regions. The action-selection cycle is also divided into two stages: comprehension of screen objects, e.g., hypertext links, and selecting the most appropriate screen object after a (semantic) evaluation. The hyperlink with the highest similarity to the goal is selected. This evaluation and selection process is repeated for every new screen page until the user reaches the target page.

Because of the basic similarities between a spatial node graph and a web graph with its node-link structure and their use, as well as the fact that both systems feature rich information spaces and encourage exploration, it can be argued that, when carrying out information-searching tasks, actions and their structure in hypertext systems are very similar to actions carried out in VR educational games that use a node-based locomotion system. In Table 1, we summarized the set of typical actions and processes in hypertext and VR systems. As indicated in the table, many actions and assumed cognitive processes are similar, just like the characteristic difficulties these processes might evoke. These involve but are not limited to: focusing on the wrong location, an overload of working memory, failures in the activation of information from long-term memory (Karanam and Van Oostendorp, 2020). In addition to this, similar to hypertext systems, in VR educational games, users have to navigate large and complex information spaces, making many decisions, to carry out and solve information-searching tasks.

**Table 1:** Similarities between navigation in hypertext and node-based VR environment.

**Typical actions and processes in hypertext systems**	**Typical actions and processes in a node-based VR game**
Parsing a page into top-level schematic regions	Parsing a visual scene into top-level schematic regions
Focus and attend to one of the schematic regions	Focus and attend a promising region
Comprehending and elaborating the screen objects (e.g., hyperlinks) within that region	Examining and elaborating items (e.g., icons and objects) within that region
Evaluating and selecting one of the screen objects	Evaluating and picking up an item
Moving on to another screen object or repeating preceding actions and processes	Moving to another node within the environment or repeating preceding actions and processes

For both node-based VR educational games and hypertext, navigation is a key issue in a learning process where information acquisition is key. Users suffering from disorientation and cognitive overload will have trouble completing information-searching tasks, leading to impaired learning performance. Therefore, based on the above analysis of the cognitive task, measures successfully designed for assessing the usability of hypertext systems (hypertext usability measures) can perhaps be used in these types of VR games using node-based movement and mimic exploration and browsing found in hypertext systems. An attractive feature of the availability of such measures is that they could result in possible real-time monitoring values for subsequently adapting a game to a player's skill level, leading to optimal learning (Shute et al., 2017). It is not our purpose to validate the task analysis above, but to provide evidence for the relevance of the navigation efficiency measures as cognition-based real-time game analytics.

### 2.2. Lostness

We pose that lostness (Smith, 1996), an unobtrusive navigational efficiency measure shown to be successful in predicting success in information-seeking tasks in hypertext, can also be used in VR games that use a node-link approach, due to the similarity in process and structure. This measure has already been shown to be effective in VR for generating navigational support as well as having a strong correlation with spatial ability (Van Oostendorp and Karanam, 2013), which is strongly linked with navigational efficiency.

The lostness measure (Smith, 1996) was created as a method for measuring hypertext usability in terms of task performance based on the belief of the author that “measures based on time and errors seem inappropriate for hypertext systems which, by their nature, encourage exploration, and browsing.” Instead, a better approach is to assess task performance in terms of the efficiency in how users find information and the degree in which they become lost in the information search whilst looking for this information (Gwizdka and Spence, 2007).

For information-searching tasks, lostness is defined by the total number of information items inspected compared with the minimum number of items that need to be inspected to locate the requested information. This leads to the following formula:

(1) $$\begin{array}{l} L=\sqrt{{\left(\frac{N}{S}-1\right)}^{2}+{\left(\frac{R}{N}-1\right)}^{2}}, \end{array}$$

where *R* is the *minimum* number of nodes needed to be visited, *S* is the *total* number of nodes a player has visited, and *N* is the number of *unique* nodes a player has visited. This will return a value between 0 and $\sqrt{2}$. 0 indicates that the task has been completed perfectly and the player was not disoriented (high navigational efficiency) and $\sqrt{2}$ indicates that a user was completely disoriented (low navigational efficiency) whilst completing the task. The *S* and *N*-values must be logged as the player is carrying out the task whilst the *R*-value must be manually specified or calculated using a path-finding algorithm. Two components are important in this formula: ($\frac{N}{S}-1$) indicating the degree of repetition to already visited nodes, and ($\frac{R}{N}-1$) indicating the degree of detours during navigation. This is a highly versatile measure that can be used in any activity that can be defined in a minimum number of steps (*R*) and the path that a user/player/person taken can be measured to provide *S* and *N*.

#### 2.2.1. Global vs. Local Lostness

In a hypertext environment, one can distinguish information-searching tasks as either *gathering* tasks or *fact-finding* tasks. Gathering tasks are defined as information searching tasks where target information spread out over different areas in the virtual space must be located and combined, whereas, fact-finding tasks are defined as tasks where required information can be located in a single, specific place (Puerta Melguizo et al., 2012).

Educational games teach content using in-game learning activities, tasks and objectives, which are based on real-life examples, and act as practice for real-life tasks (Lester et al., 2018). In a game, a player can be given a task to complete, such as finding three items that are related to a certain topic. Each of these items can be regarded as an objective. To complete a task, a player must achieve all of the objectives. As such, we regard tasks and objectives as representing these in-game learning activities, in which tasks have a larger scope, where “global” gathering activities are needed, as they consist of finding information that needs to be gathered together, while finding the separate objectives represent “local” fact-finding activities, which have a smaller scope.

Based on these two types of tasks, two corresponding versions of the lostness measure were defined: *global lostness*, which focuses on tasks, and *local lostness*, which focuses on objectives. This allows information-searching tasks to be measured from both a “global” task-based perspective and a more “local” objective-based perspective.

*Global lostness*: Global lostness focuses on tasks, using the initial lostness equation to give a value of lostness from the start of each task until it is completed. A weighted lostness (*L*) mean based on the number of objectives per task (*x*), reflecting the complexity of each task (*t*), is then used to give a measure of lostness for the full game (*L*_*G*_):

(2) $$\begin{array}{l} L_{G}=\frac{\sum_{t=1}^{n}\left(L_{t}\times x_{t}\right)}{\sum_{t=1}^{n}x_{t}}, \end{array}$$

In this measure, the *S* and *N* values are logged from the moment a task is started up until it is completed. The *R*-value is often manually inputted based on data from a perfect playthrough of the game. To give a more accurate overview of the player performance over the full game, a weighted mean is used rather than an ordinary mean, as tasks with more objectives require more searching than shorter tasks to access the learning content, resulting in longer paths being followed. Consequently, lostness could be overstated by low lostness values from simpler tasks masking higher lostness values from more complicated tasks and vice-versa.

*Local lostness*: Local lostness focuses on objectives, regardless of the task that they belong to. As well as calculating a lostness value for each objective, using the initial lostness equation, the *R*, *S*, and *N*-values of each objective (*O*) are summed to give a measure of local lostness for the full game (*L*_*L*_):

(3) $$\begin{array}{l} L_{L}=\sqrt{{\left(\frac{\sum_{o=1}^{n}N_{o}}{\sum_{o=1}^{n}S_{o}}-1\right)}^{2}+{\left(\frac{\sum_{o=1}^{n}R_{o}}{\sum_{o=1}^{n}N_{o}}-1\right)}^{2}}, \end{array}$$

In this measure, the *S* and *N*-values are always being logged from the start of the game and are reset to 0 after an objective has been completed. Because there are probably multiple orders in which objectives can be completed, a path-finding algorithm, such as a breadth-first search algorithm, is usually necessary to calculate the shortest path (*R*) between objectives.

Rather than only focusing on a limited set of objectives that belong to a task, local lostness considers all objectives in the game. This is because it is considered a mistake to make a game linear when it aims to tell a story and make the player part of it (Hudson, 2011). This also has the additional benefit of including objectives that act as task starting points, which also must be searched for and may contain educational content. As such, this measure encompasses all objectives, which must be completed. Therefore, local lostness can follow the complete path of the player through the game. The *R*, *S*, and *N*-values from each objective are fed into the original lostness formula to give a full game local lostness measure *L*_*L*_, which reflects the full path taken by a player throughout the whole game, essentially treating the whole game as one long task.

See Supplementary Materials for more detailed implementations, algorithms, and examples of both lostness measures being used.

### 2.3. Potential Other Relevant Factors to Learning in VR

Although it is proposed that measuring disorientation/navigational efficiency is important when the goal of a game is information acquisition to meet learning goals, the type of motivational/emotional experience that a player has whilst playing these kinds of educational games should also be taken into account (Lee et al., 2010). This is backed up by research showing that cognitive and emotional aspects of player involvement with the game environment, particularly enjoyment and deep thinking, also positively influence information processing (Imlig-Iten and Petko, 2018).

Three elements of player experience are thought to be noteworthy when it comes to having a positive effect on learning in VR: (a) presence, i.e., the experience of being integrated into a mediated environment and the sense of being there (Slater, 2002), (b) engagement, i.e., heightened concentration, involvement, and enjoyment (Kim, 2018), and (c) cognitive interest i.e., understanding key topics presented by the game and becoming more interested (Harp and Mayer, 1997).

In many studies, engagement is seen as an important effect of games (and determinant of game playing) and recent research has concluded that both emotional and cognitive engagement catalyze learning in games (Boyle et al., 2012). Furthermore, presence is viewed as being of particular importance in VR games, a medium where increased presence is a defining feature (Steuer, 1992), and was found to aid learning outcomes (Lee et al., 2010). Finally, cognitive interest serves as intrinsic motivation to explore and experience new and unfamiliar things (Van der Sluis et al., 2014), a key part of VR educational games.

For all three aspects, we assume, based on empirical studies (Boyle et al., 2012; Abdul Jabbar and Felicia, 2015), that they are positively influenced by a (VR) game environment and are positively related to learning. Accordingly, we will measure these game experience aspects through three questionnaires that have proven validity: the I-group Presence Questionnaire (Schubert, 2003), the Game Engagement Questionnaire (Brockmyer et al., 2009), and the Perceived Interest Questionnaire (Schraw et al., 1995) to investigate the experience aspects of a player whilst playing the game.

## 3. Research Questions

As mentioned, navigation is key when it comes to information searching to achieve learning goals and disoriented players have trouble completing tasks and objectives, which we expect to have a negative effect on learning. In this regard, lostness is a process-oriented measure of what participants are learning. We expect that a higher performance in navigation should contribute to learning. In other words, if a player is efficient, they should learn better. Accordingly, we will apply and evaluate the lostness hypertext usability measure in an appropriate VR game to measure navigational efficiency and identify this disorientation. Two related measures, “global” and “local” lostness, stemming from the type of information-searching tasks identified by Puerta Melguizo et al. (2012), will be investigated to examine if they provide an online indication of how well players learn from a VR educational game.

Secondly, because we may assume that more factors than lostness will be active, such as the game experience aspects of presence, engagement, and cognitive interest, which have been linked to learning, we will also investigate whether the effect of these game experience aspects influence learning.

Therefore, we will compute the correlations of both lostness measures (global and local) with experience aspects (cognitive interest, engagement, and presence) as well as with learning by participants. This will allow us to examine the effect of both lostness measures as well as of these three additional player experience aspects *on* learning. This brings us to the following research questions: In a VR educational game, when it comes to learning, what is the effect of:

- Global lostness and local lostness?
- Experience aspects? (i.e., presence, engagement, cognitive interest)

Because a low lostness score denotes that the participant followed an efficient (perfect in the case of a value of zero) search path and a high score represents a chaotic, imperfect search, a lower lostness value indicates higher navigational efficiency and less disorientation. Consequently, we predict that:

- There will be a negative correlation between learning and both global and local lostness.
- There will a positive correlation between learning and the game experience aspects of engagement, presence, and cognitive interest.

The effect of the lostness measures on learning will be analyzed via three steps (using IBM *SPSS* Statistics 24). First, all measures will be investigated through their means and standard deviations (SDs). Next, we carry out a zero-order correlation analysis, which will show if these measures give any kind of indication of whether or not the measures are related to one another. Finally, the contribution of the lostness measures (global and local) and experience aspects (presence, cognitive interest, and engagement) to learning performance will be investigated in multiple linear regression analyses to give a more pure estimation of the effect of lostness on learning when the experience aspects are taken into account.

## 4. Materials and Methods

### 4.1. Apparatus

#### 4.1.1. Game

Sony PlayStation VR game “The Chantry,” a VR educational game using a node-based movement system was used. This game takes place in the house of Dr. Edward Jenner and tells the story of the invention of the smallpox vaccine https://jennermuseum.com/.

**Figure 3:**
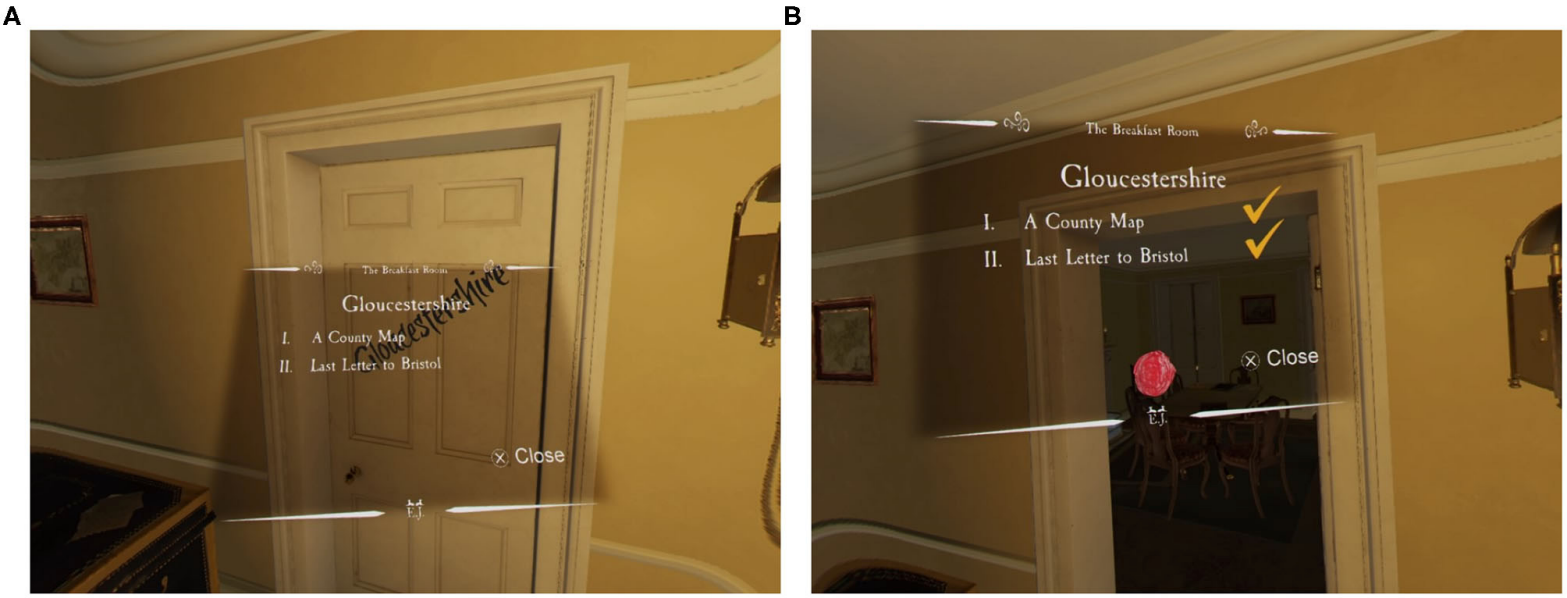
The list of objectives shown in *the Chantry* when the “*Gloucestershire*” task is started **(A)** and the completed list **(B)**, with all objectives ticked off and a wax seal applied.

**Figure 4:**
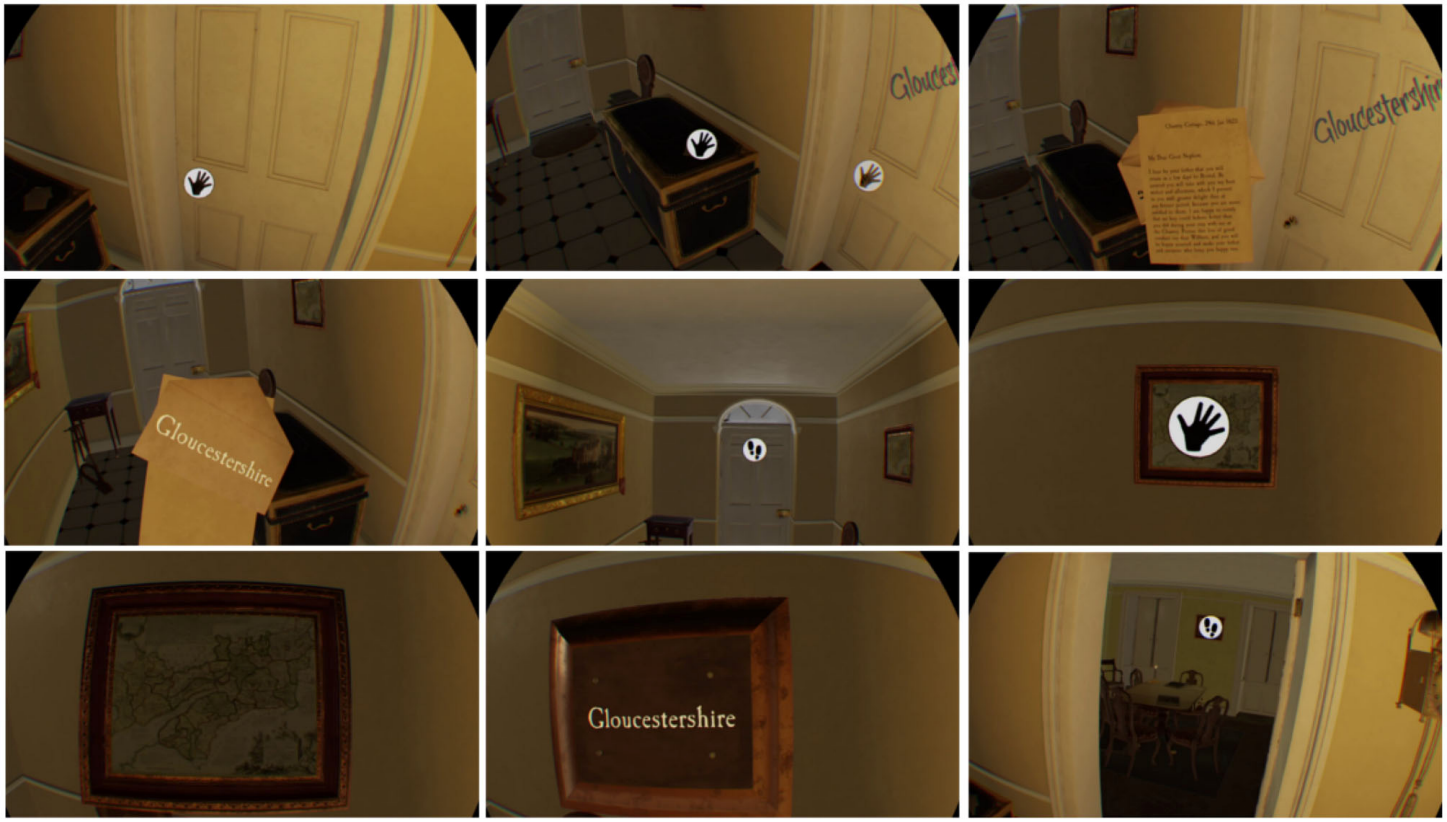
The actions taken to complete the “*Gloucestershire*” task in “*the Chantry*.” The door is closed until the player navigates toward two items, picks them up, and turns them over to reveal the word on the back.

To progress through games such as “The Chantry,” players interact with closed doors and window shutters. Upon trying to open these, a word appears (e.g., “*Gloucestershire*” in Figure 3), which represents an educational story topic, and an audio narrative is then played, containing story information that the player needs to learn about. The player is then presented with a list of objectives e.g., “*County Map*” and “*Last Letter to Bristol*” in Figure 3) with the same at the top as on the door/shutter (e.g., “*Gloucestershire*” in Figure 3). Each objective in the list (e.g., “*County Map*” and “*Last Letter to Bristol*” in Figure 3) is a hint toward an item that must be found. The player must navigate the virtual environment and find the item that the objective is referring to and turn it over in their hands to reveal the same keyword that is written on the door. When one of these items is found, completing the objective, the items reveal additional story information (fact-finding task) in the form of an audio narrative. Achieving all of the objectives completes the task, opening the door/shutter, and another audio narrative plays, revealing additional story information, together forming an information-searching (gathering) task. See Figure 4 for an example of a task (“*Gloucestershire*” as shown in Figure 3) being carried out in *the Chantry*.

In this kind of in-game learning activities, there is always an efficiency aspect, i.e., a shorter path or sequence of nodes that must be visited to complete an objective and, therefore, task. This makes the lostness measure suitable here. See Supplementary Materials for how both lostness measures are applied.

#### 4.1.2. Hardware

Each participant wore a Sony PlayStation VR headset (model: CUH-ZVR1) to play the game on a Base PlayStation 4 Development Kit and, to realize maximum comfort, all participants wore their own over-ear headphones for audio. This accurately replicates how VR games are played with complete immersion in the VR environment without distraction, as the participants could only see and hear what was happening in the game. They also made use of the standard DualShock 4 controller (model: CUH-ZCT1). To control the game, participants used their head movements to look at a node and used a single button press on the controller to move to that node or pick up an item. Once picked up, an item is moved and rotated by doing the same action holding on the controller.

### 4.2. Measurements

Each interaction participants carried out, including jumping to another node, picking up and examining an item, and unlocking something, was captured. As this data was timestamped, it was possible to trace the path that has been taken and calculate the lostness measures.

Knowledge retention was measured through a bespoke knowledge test, consisting of 24 randomized true/false statements (50% true and 50% false). Sixteen of these questions concerned facts, relating to the story (e.g., “Vaccination was already popular in England by 1,800”), and 8 involved spatial aspects, relating to the location of items/rooms in the game (e.g., “The library was very close to the dining room and located on the first floor”).

Standard questionnaires, consisting of five-point Likert scales, were used to measure engagement, presence, and cognitive interest. These were: the Game Engagement Questionnaire (Brockmyer et al., 2009) (example item—“I feel like I can't stop playing”), the igroup Presence Questionnaire (Schubert, 2003) (example item—“I felt present in the virtual space”), and the Perceived Interest Questionnaire (Schraw et al., 1995) (example item—“I thought the game's topic was fascinating”). Each of these questionnaires consists of 5-point Likert scales, with high Cronbach's coefficients-alpha, being respectively 0.85, 0.85, and 0.90.

### 4.3. Procedure

Upon being seated, participants were given health and safety information, which consisted of warnings about possible motion sickness, advice to withdraw if it occurs, and to remain seated and not attempt to physically interact with the virtual environment. both in oral and written form. They were also given instructions on how to play the game. Also, an informed consent form was given. They were asked if everything was clear to them. If not, an additional explanation was provided. After this, they signed the informed consent form. The participants were informed that they would be playing a VR educational game and would be tested after playing to investigate how much knowledge they were able to recall. They were instructed to divide their attention to the whole environment and not to stick to one location. They were then given 30 min to play through the game at their own pace, learning about the story, and completing tasks and objectives. The tasks and objectives in the game, varying in complexity, were the same for all participants and presented in the same order. To control the game, players used their head movements to look at a node and used a single button press on the controller to move to that node or pick up an item, which is moved and rotated by doing the same action whilst holding the controller. Once the participants had played the game for the full 30 min, they immediately completed the knowledge test and the game experience questionnaires. Finally, the participants were debriefed and informed about the nature of both the study and the game.

## 5. Initial Study

### 5.1. Participants

A total of 13 students aged 13–18 (mean: 15, SD: 1.354), 6 males and 7 females, from a University Technical College in England, with differing levels of experience in using VR were selected. They were only permitted to participate if they did not suffer from epilepsy, migraines, or motion sickness when in moving vehicles, in line with recommendations on the use of VR (Sony Interactive Entertainment LLC, 2020).

### 5.2. Results

#### 5.2.1. Means and Standard Deviations

Firstly, the means and standard deviations (SDs) of all measures (global lostness, local lostness, presence, cognitive interest, and engagement) were compared across participants. The outcomes were as follows (see Table 2):

**Table 2:** Lostness, knowledge, and in-game experience questionnaire means and standard deviations from the first study.

	**Mean**	**Standard deviation**
Global lostness	0.571	0.147
Local lostness	0.464	0.112
Knowledge test (Proportion correct)	0.590	0.147
Cognitive interest	3.654	0.521
Engagement	2.915	0.515
Presence	3.005	0.350

This shows that participants were only moderately effective at the in-game tasks, as the average of both lostness measures was between 0.40 and 0.60; local lostness was 0.464 (SD: 0.112) and global lostness was 0.571 (SD: 0.147). The difference between the global lostness and local lostness is considerable [*t*_(12)_ = 4.474, *p* < 0.001] and global lostness is, in general, higher than the local lostness. Therefore, “globally” players are more lost than “locally.” Despite this, as expected, both lostness measures correlated significantly (0.811, *p* < 0.001).

When it comes to the knowledge test, the mean proportion of correct items on the overall knowledge test was 0.590 (SD: 0.147), somewhat above the chance level. This is in line with the spatial questions, which were only answered slightly better, with a mean of 0.615 (SD: 0.173).

For experience aspects, the results were moderately positive, as they were around or above the mid-point of the five-point scale. The mean cognitive interest was 3.654 (SD: 0.521), engagement was 2.915 (SD: 0.515), and presence 3.005 (SD: 0.350).

#### 5.2.2. Zero-Order Correlations

The main research question was to investigate if the lostness and game experience measures indicate how well the player learns from a game. To begin with, the zero-order correlations are presented in Table 3.

**Table 3:** Zero-order correlations between knowledge, lostness, and experience measures from the first study.

	**Global lostness**	**Local lostness**	**Cognitive interest**	**Engagement**	**Presence**
Knowledge test	−0.435	−0.643^*^	0.163	0.054	−0.597^*^
Global lostness	–	0.811^**^	−0.288	−0.118	−0.059
Local lostness		–	−0.023	0.067	0.132
Cognitive interest			–	0.440	0.093
Engagement				–	0.050

* *p < 0.05*,

** *p < 0.001*.

The results of this correlation analysis show that both lostness measures correlate negatively with learning, as is expected, although only the correlation with local lostness is significant (*p* = 0.018) whereas global lostness is not significant (*p* = 0.138). This strong significant (negative) correlation gives weight to the assumption that local lostness can be used as a game analytic measure.

Although the cognitive interest and engagement measures did not show a significant correlation with the knowledge test results, there is, interestingly, a negative significant correlation (*p* = 0.031) between presence and the knowledge test results. This showed that participants learned less the more present they felt.

#### 5.2.3. Multiple Linear Regression Analyses

The main analysis of the current study is to examine whether lostness measures and player experience aspects are related to knowledge acquisition (i.e., performance on the knowledge test). To investigate this, statistical analyses were performed in which the relationship between the final knowledge test as criterion variable and both lostness measures, as well as cognitive interest, engagement, and presence, are computed as predictors in regression analyses. This statistical technique enables us to examine the relative influence of variables (the predictors) corrected for the influence of the other variables on the criterion variable, the knowledge test.

We conducted multiple linear regression analyses with all these variables, applying the ENTER method offered by *SPSS*. We checked the normality distributions of the variables and their error distributions, using the Shapiro-Wilk test, and found that two variables (spatial knowledge and cognitive interest) deviated significantly from normality (*p* < 0.05). However, regression analysis has been shown to be quite robust even when the normality assumption is violated (Hair et al., 2009, p. 197). Some scholars even claim that normality is not demanded at all (Lumley et al., 2002; Schmidt and Finan, 2018). This seems to hold particularly with larger samples. In this study, due to practical constraints, only 13 participants were observed. Further, we checked the collinearity of the independent (predictor) variables. In all cases the conditions of non-collinearity were satisfied (*p* < 0.05), based on the Variance Inflation Factor values.

The results of the multiple linear regression analyses are shown in Table 4.

**Table 4:** *Beta-coefficients* between knowledge, lostness, and experience measures from the first study.

	***Beta-coefficient* overall knowledge**	***Beta-coefficient* spatial knowledge**
Global lostness	0.213	0.598
Local lostness	−0.746 ^(*)^	−0.832^*^
Presence	−0.510^*^	−0.641^*^
Cognitive interest	0.231	0.175
Engagement	0.052	−0.058

(*) *0.05 < p < 0.10*,

* *p < 0.05*.

Multiple *R* = 0.855, *F*_(5, 12)_ = 3.821, *p* = 0.055 for Overall Knowledge;

Multiple *R* = 0.90, *F*_(5, 12)_ = 5.94, *p* = 0.019 for Spatial Knowledge.

The multiple correlation coefficient concerning overall knowledge is 0.855, thus explaining 73% of the variance on the knowledge test. The main contributing variables, reported in Table 4, are local lostness (*Beta-coefficient* = −0.746) and presence (*Beta-coefficient* = −0.510) with presence being significant (*p* = 0.043) and local lostness showing a trend (*p* = 0.092). Cognitive interest and engagement did not appear to be relevant.

We also checked separately which variables explain spatial knowledge acquisition: we find a multiple correlation coefficient of 0.90, explaining 81% of the variance of the spatial knowledge test. Local lostness (*Beta-coefficient* = −0.832, *p* = 0.037) and presence (*Beta-coefficient* = −0.641, *p* = 0.008) appeared to be the strongest predictors with presence being significant (*p* = 0.008) along with local lostness (*p* = 0.037). Global lostness, cognitive interest, and engagement were not found to have any significant effect.

## 6. Expanded Sample from a Second Related Study

After the first study had been completed, a second empirical study was carried out with the same game, which examined different research questions. It investigated the effect of interaction and story structure on learning (Ferguson et al., 2020a). However, as the control condition in that study followed the same methodology as our first study (same game, same VR equipment, same measurements, same instruction, similar participants), these participants can be combined with the previous participants, to get a more reliable indication of the predictive validity of the lostness measure.

### 6.1. Participants

As with the first study, a total of 12 students aged 13–18 (mean: 15.67, SD: 1.231), 10 males and 2 females, were selected from a University Technical College in England, with differing levels of experience in using VR were selected. Again, they were informed that they would be playing a VR educational game and would be tested after playing to investigate how much knowledge they were able to recall.

One (female) participant's results were ultimately excluded from the results for being an outlier, having a local lostness value of more than two standard deviations above the mean (Stevens, 1999). Moreover, this participant struggled to play the game compared to other participants and did not make much progress, taking too much time to the beginning of the game and not accessing the educational content to be learned. This left 11 participants (mean age: 15.25, SD: 1.294, 16 males, 8 females). These 11 participants were combined with the 13 participants from the previous study, giving a total of 24.

### 6.2. Results

#### 6.2.1. Means and Standard Deviations

Once again, the means and standard deviations of all measures (global lostness, local lostness, presence, cognitive interest, and engagement) were compared across participants. The outcomes were as follows (see Table 5):

**Table 5:** Lostness, knowledge, and in-game experience questionnaire means and standard deviations from the combined studies.

	**Mean**	**SD**
Global lostness	0.605	0.166
Local lostness	0.484	0.102
Knowledge test (proportion correct)	0.576	0.119
Cognitive interest	3.667	0.693
Engagement	2.976	0.563
Presence	3.179	0.450

Once again, this shows that participants were only moderately effective at the in-game tasks, as the average of both lostness measures was 0.484 (SD: 0.102) and 0.605 (SD: 0.166), respectively for local and global lostness. The difference between the global lostness and local lostness is considerable [*t*_(23)_ = 4.789, *p* < 0.001]. Once again, global lostness is higher than the local lostness, strengthening the claim that players are more lost “globally” than “locally.” As before, both lostness measures correlated significantly (0.669, *p* < 0.001).

When it comes to the knowledge test, the mean proportion of correct items on the overall knowledge test was 0.576 (SD: 0.119), also somewhat above the chance level yet marginally lower than the results from the first study (−0.014). Compared to the first study, when focusing on the spatial questions, these were answered slightly worse, with a mean of 0.563 (SD: 0.165). So, both overall and spatial knowledge were roughly in line with each other.

For experience aspects, the results were moderately positive, and, as with the results of the first study were around or above the midpoint of the five-point scale. The mean of cognitive interest was 3.667 (SD:0.693), engagement was 2.976 (SD: 0.563), and presence 3.179 (SD: 0.450).

#### 6.2.2. Zero-Order Correlations

Again, but with a larger dataset, we investigated if the lostness and game experience measures indicate how well the player learns from a game. The zero-order correlations are presented in Table 6.

**Table 6:** Zero-order correlations between knowledge, lostness, and experience measures from the combined studies.

	**Global lostness**	**Local lostness**	**Cognitive interest**	**Engagement**	**Presence**
Knowledge test	−0.444^*^	−0.597^**^	0.377^*^	0.160	−0.268
Global lostness	–	0.669^**^	−0.382^*^	−0.251	0.109
Local lostness		–	−0.197	0.031	0.031
Cognitive interest			–	0.525^**^	0.251
Engagement				–	0.263

* *p < 0.05*,

** *p < 0.001*.

Once again, the results of this correlation analyses show that both lostness measures correlate negatively with learning, yet this time the correlation is significant for global lostness (*p* = 0.015) and highly significant for local lostness (*p* = 0.001). These strong significant (negative) correlations give weight to the assumption that both lostness measures can be used as a game analytic measure yet local lostness is a better measure to use.

Unlike the first dataset, cognitive interest significantly correlated with the knowledge test results (*p* = 0.035) showing that participants learned more the more interested they were. Cognitive interest also significantly correlated with engagement (*p* = 0.004) and global lostness (*p* = 0.033). There was no correlation between the knowledge test results and presence, unlike with the first dataset or, once again, engagement.

#### 6.2.3. Multiple Linear Regression Analyses

Once again, a regression analysis is carried out to examine whether the lostness measures and player experience aspects are related to knowledge acquisition (i.e., performance on the knowledge test).

We checked anew the normality distributions of the dependent variables and their error distributions, using the Shapiro-Wilk test, and found the same variables, spatial knowledge and cognitive interest, still deviated from normality (*p* < 0.05), see the earlier remark on this in section 5.2.3. Once again, in all cases the conditions of non-collinearity were satisfied (*p* < 0.05), based on the Variance Inflation Factor values.

The results of the multiple linear regression analyses are shown in Table 7.

**Table 7:** *Beta-coefficients* between knowledge, lostness, and experience measures from the combined studies.

	***Beta-coefficient* overall knowledge**	***Beta-coefficient* spatial knowledge**
Global lostness	0.217	0.476
Local lostness	−0.667^**^	−0.629^*^
Presence	−0.399^*^	−0.473^*^
Cognitive interest	0.346 ^(*)^	0.255
Engagement	0.158	0.042

(^*^) *0.05 < p < 0.10*,

* *p < 0.05*,

** *p < 0.01*.

Multiple *R* = 0.752, *F*_(5, 23)_ = 4.676, *p* = 0.007 for Overall Knowledge;

Multiple *R* = 0.617, *F*_(5, 23)_ = 2.213, *p* = 0.098 for Spatial Knowledge.

In this dataset, the multiple correlation coefficient concerning overall knowledge is 0.752, thus explaining 56.6% of the variance on the knowledge test, compared to the 73% found in the first study. The main contributing variables, reported in Table 7, are local lostness (*Beta-coefficient* = −0.667, *p* = 0.007), presence (*Beta-coefficient* = −0.399, *p* = 0.030), and cognitive interest (*Beta-coefficient* = 0.346, *p* = 0.094). Again, engagement did not appear to be relevant. Interestingly, despite the significant correlation between global lostness and the knowledge test, this was not a significantly contributing variable. This gives further weight to the assumption that local lostness is the better measure for predicting learning.

We also checked separately which variables explain spatial knowledge acquisition: we find a weakly significant multiple correlation coefficient of 0.617, explaining 38.1% of the variance on the spatial knowledge test. Once again, local lostness (*Beta-coefficient* = −0.629, *p* = 0.028) and presence (*Beta-coefficient* = −0.473, *p* = 0.031) appeared to be the strongest and significant predictors. Global lostness, cognitive interest, and engagement were not found to have any significant effect.

## 7. Discussion

Altogether, two different novel unobtrusive and implicit hypertext navigational efficiency measures (local and global lostness) and experience aspects (presence, engagement, and cognitive interest) were examined in a VR educational game employing a node-based movement system. Learning was measured by a post-game knowledge test consisting of factual and spatial information items. We will first briefly summarize the zero-order correlations. Using a small sample (12 participants) from an initial study (Ferguson et al., 2020b), we found a negative correlation of both navigation efficiency measures with performance on a knowledge test and, therefore, how well the player learned from the game. However, only the correlation with local lostness was significant. A negative significant correlation was also observed between presence and how well the player has learned from the game, the opposite of what was expected. No significant correlations were found for engagement and cognitive interest. The sample size was subsequently expanded (24 participants) by including data from the control condition from a second study (Ferguson et al., 2020a). When examining this larger sample size, we found significant negative correlations with the knowledge test for both lostness measures, confirming Hypothesis I, with strong support particularly for local lostness. Regarding the experience aspects, again, there was no significant correlation of engagement with the knowledge test results, yet a significant correlation was found for cognitive interest. However, the significant negative correlation between presence and knowledge test results from the first study was not present in the larger dataset. Nevertheless, both sets of data led to a partial rejection of Hypothesis II, because only cognitive interest correlates positively with learning while presence in Study 1 correlates even negatively.

Interestingly, cognitive interest was also found to significantly and negatively correlate with global lostness. Participants with higher interest for the key topics presented by the game show higher global navigation efficiency. Apparently cognitive interest is positively associated with global navigation efficiency, and it is worthwhile to note that both correlate positively with knowledge acquisition in the extended study. It must also be addressed that there was no significant effect between engagement and learning, although there is a highly significant correlation between engagement and cognitive interest, which have previously been found to be positively related when it comes to learning (Mazer, 2013). This lack of correlation may be related to the fact that the Game Engagement Questionnaire (GEQ) was developed considering video game violence (Brockmyer et al., 2009) so may not be appropriate to our learning context. Other research also notes that multiple measures of engagement should be used, particularly physiological measurements (Appleton et al., 2006; Boyle et al., 2011). It could be that the use of a different questionnaire, such as the User Experience Scale (Wiebe et al., 2014) or these additional measures would reveal an effect of engagement on learning.

Of more importance than the above correlations are the *Beta-coefficients* from the multiple linear regression analyses. These outcomes enable us to get an estimate of the influence of the predictor variables when they are corrected for the influence of the other predictor variables. Furthermore, they inform us of the relative contribution of the respective predictor variables to the variance in the criterion variable.

Starting with the lostness measures, in the smaller sample and examining the effect of each of the independent variables on the results of the knowledge test, whilst taking all of the other variables into account, we find a weakly significant negative *Beta-coefficient* between local lostness and the overall knowledge test results and a significant *Beta-coefficient* when focusing on the spatial aspects on the knowledge test. When carrying out the same analyses in the larger sample, we again find, for local lostness, negative *Beta-coefficients*, this time highly significant for overall knowledge and significant for spatial knowledge. Global lostness has no significant *Beta-coefficients* in either of the samples. Therefore, a higher local lostness score, that is, when a player has low navigational efficiency and is disoriented when navigating an environment, with more detours and revisits, apparently leads to less knowledge acquisition. Conversely, a player with high navigational efficiency (not disoriented) will learn better. This is evidenced by the highly significant negative correlation and *Beta-coefficients*. Thus, it appears to indicate that local lostness measures how well players process information and that high local lostness indicates difficulties in this information processing and cognitive overload (Gwizdka and Spence, 2007).

When it comes to the experience aspects, in the smaller sample, no significant *Beta-coefficient* was found between both engagement and cognitive interest and learning, although a significant negative *Beta-coefficient* was found between presence and both overall knowledge and spatial knowledge. This result is repeated in the larger sample. These results indicate that, for both overall and spatial knowledge, a person learns less the more present they feel. Slightly different results are found in the larger sample for the other two experience aspects. There was a weakly significant *Beta-coefficient* of cognitive interest with overall knowledge, backing up previous findings (Van der Sluis et al., 2014). The finding that lostness predicts learning performance is expected and aligns with cognitive load theory, which is expected since disorientation, measured by lostness, is known to invoke cognitive overload and have an adverse effect on learning. In contrast, it was expected that any effect of presence on learning would be positive rather than negative which was the case in both studies. Research has found that building a spatial mental model of a situation is expected to lead to a sense of spatial presence (Bailey and Witmer, 1994; Lee et al., 2010). However, other recent research was unable to find a significant relationship (Alsina-Jurnet and Gutiérrez-Maldonado, 2010; Ling et al., 2013; Coxon et al., 2016). It could be that the additional feeling of presence involves cognitive overload. Recent studies have shown that the higher immersion of virtual reality and higher presence can lead to higher cognitive load, resulting in decreased learning (Schrader and Bastiaens, 2012; Makransky et al., 2019; Frederiksen et al., 2020). However, it must be noted that, for both samples, there was no significant correlation between presence and either of the lostness measures. This would indicate that presence is increasing cognitive load independently to lostness. Altogether, in the literature, there are three types of cognitive load identified: intrinsic, which is associated with the complexity of the task, extraneous, which is related to the way that the task is presented, and germane, which is produced by processing and constructing schemas to handle new information for learning new skills (Sweller et al., 2011). Intrinsic and germane cognitive load are an essential part of the learning process, and it can be assumed that lostness represents extraneous cognitive load, as it is related to the presentation of the task itself. Increased presence, in the form of enhanced visual information provided by VR, could also be leading to increased extraneous cognitive load. This could be an inherent weakness in the use of VR for learning. It could even point toward research stressing that educational games should be designed differently to better make use of VR's advantages when transferring information. Recent research suggests that communicating information visually is preferable when using VR, making the most of its prominent modality (Huang et al., 2019).

Proposals to avoid the problem of cognitive overload, based on cognitive load theory have been both successful and unsuccessful (Andersen et al., 2016a,b). The negative effect that presence has on learning suggests that reducing extraneous cognitive load could increase learning, something often suggested (Sweller et al., 1998). In this respect, the effect of lostness on learning shows that this measure could be used to adapt the game difficulty to the proficiency of players to assist them before cognitive load is too high, players become too overloaded and their learning is negatively affected. Adaptation would reduce extraneous load and would build on our previous research using the same game (Ferguson et al., 2020a), which showed that participants learned significantly more when they were guided through the environment at the expense of a lesser feeling of cognitive interest and presence. Thus, it should be investigated if aiding players when they are identified as being disoriented can lead to the same high levels of learning, by reducing cognitive load, without negatively affecting the experience players felt they had in the game. The reduced extraneous cognitive load may free up cognitive capacity so that extraneous cognitive load caused by presence does not have an impact on learning, further enhancing the experience whilst learning. This will be studied as a priority in our future research. Further research must also be carried out to determine whether the measure can be generalized to other types and genres of games, in other areas, or using other locomotion techniques and different activities.

It must be addressed that there is a strong and highly significant correlation between global and local lostness in both the smaller and larger dataset. This is expected as both measures are different interpretations of lostness, and both have overlapping activities and make use of the same original lostness formula. Yet the constraint of non-collinearity was satisfied. Non-collinearity means the amount of variability of an independent (predictor) variable not explained by the other independent variables (Hair et al., 2009). In other words, when the non-collinearity is high, the independent variables contribute sufficient unique variance. Although global lostness had similar correlations as local lostness, the *Beta-coefficients* with learning, both overall and spatially, are different and for local lostness significant but not for global lostness, showing that local lostness is, empirically, a better measure.

Educational narrative games, by nature, encourage exploration. Players are more inclined to look around and encounter different tasks and objectives, likely carrying out multiple tasks simultaneously. This comes back to the point that games are not always linear (Hudson, 2011). Local lostness is more appropriate for these types of games as it considers all possible objectives. Conversely, global lostness considers each task independently, giving a value of lostness from the start of each task until the end. Thus, some players may be incorrectly regarded as lost, because they are carrying out more than the minimum number of steps and revisiting different nodes for each task, even though they are completing other necessary tasks and objectives. This is especially true when one task may rely on another task or objective being completed before all task objectives become available. This could be a major reason for the lack of a significant *Beta-coefficient* of global lostness with learning. Another reason for this low *Beta-coefficient* could be that the knowledge test used was not assessing the gathering aspect of information processing by consisting of questions regarding isolated facts. This would make it more suitable for fact-finding tasks and, thus, local lostness. Perhaps having questions more suited to gathering activities, such as mentioning different objectives related to a task, would be more appropriate when using global lostness. Further research is needed to examine this issue. All in all, based on empirical as well of theoretical considerations, local lostness should be preferred as an online implicit (real-time) measure for learning in VR educational games.

## 8. Conclusion

Overall, it was shown in this study that an implicit and unobtrusive hypertext usability measure has high real-time predictive validity when applied to a certain genre of educational VR games. This applies specifically for games where information-searching activities, involving navigation, are an intrinsic part of the learning process and node-based movement is used as the locomotion system. Besides, we also identified another variable, presence, which seems to indicate, given the significant negative *Beta-coefficient* with the knowledge test, that participants get more overloaded as they feel more present and immersed. These results also confirmed the importance of cognitive interest, showing that a person learns more if they are more interested in the topic.

Overall, this opens the door for the local lostness measure to be meaningfully used as an implicit online game analytic in a dynamic adaptive system, offering assistance to players before they suffer from cognitive overload in educational games. This would offer new opportunities for on-line, in-game adaptivity dependent on user performance during play (Alsina-Jurnet and Gutiérrez-Maldonado, 2010; Shute et al., 2017). Perhaps this player-dependent adaptivity can support the cognitive processing of educational materials and prevent overload at the right time, resulting in more effective learning (Salzman et al., 1999; Lee et al., 2010).

## Data Availability Statement

The raw data supporting the conclusions of this article will be made available by the authors, without undue reservation.

## Ethics Statement

The studies involving human participants were reviewed and approved by the Sheffield Hallam University Review Ethics Committee (SHUREC). Written informed consent to participate in this study was provided by the participants, and where necessary, the participants' legal guardian/next of kin.

## Author Contributions

CF wrote the manuscript, designed and conducted the experimental study, and analyzed the data. HvO co-wrote the manuscript and oversaw the project. All authors contributed to the article and approved the submitted version.

## Conflict of Interest

The authors declare that the research was conducted in the absence of any commercial or financial relationships that could be construed as a potential conflict of interest.
