# Supplementary material for: Lost in Learning: Hypertext Navigational Efficiency Measures Are Valid for Predicting Learning in Virtual Reality Educational Games
Source: Front Psychol. 2020 Nov 25;11:578154. doi: 10.3389/fpsyg.2020.578154 (PMC7723827; doi:10.3389/fpsyg.2020.578154)
Supplement: Supplementary file 1 [file Data_Sheet_1.PDF]

frontiers

Supplementary Material

1 SUPPLEMENTARY ALGORITHMS

As alternatives to the lostness (L) equations presented in the manuscript, we provide algorithms showing how global lostness (L_G) and local lostness (L_L) are calculated within a game, including the calculations of minimum steps (R), total steps (S), and unique steps (N).

Algorithm 1: Global Lostness update when a player moves

```
Given the current player location C and a list of tasks T, with objectives T_O, player path since the start
  of the task T_P, and minimum steps T_R
foreach t ∈ T do
    if t.isActive() then
        t_P = append(t_P, C) ;                                  // Update the player path
        if t_O.contains(C) then
            t_OP.setFound(true) ;    // If at objective location, set it to found
            if t.getProgress() = 1 then
                // If task is complete, calculate lostness
                N = unique(t_P) ;                       // Total and unique steps from path
                S = length(t_P);
                t_L = sqrt((N/S - 1)^2 + (t_R/N - 1)^2);
                t.setActive(false) ;                      // Completed so no longer active
    else
        if C = t.start() then
            t.setStarted(true) ;                                      // Also sets active
    end
end
// Get lostness for the full game using a weighted mean
Function GetFullGameLostness():
    foreach t ∈ T do
        if t.isStarted() then
            L += (t_L * length(t_O));
            div += length(t_O);
        end
    end
return L/div
```

As shown in Algorithm 1, global lostness gives a value of lostness for each task. These lostness values are then used to give a global lostness value for the full game. By considering the number of objectives in each task, as part of a weighted average, this full game lostness value reflects the complexity of each task.

Algorithm 1 shows that, when a player moves, the path, containing unique and total steps, is updated for each task. This shows that each task is treated independently. If the current location contains an objective that serves as a task starting point, the associated task is started and set to active. Otherwise, an objective is found, it is marked as such within one of the active tasks and the task progress (a value between 0 and 1) is updated. Once progress has reached 1, the task is set to completed. The minimum number of steps (R), stored within the task, is used, along with the task path for S and N, to calculate lostness.

Algorithm 2: Local Lostness update when a player moves

Given a list of objectives O, the player's path since their last target P (starting location P_0), and the current player location C

```
P = append(P, C) ;                                  // Update the player path
if O.contains(C) then
    // If current location is a target, calculate lostness
    N = unique(P) ;                        // Total and unique steps from path
    S = length(P);
    R = length(shortestPath(P_0, C)) ;                  // Breadth-first search
    L = sqrt((N/S - 1)^2 + (R/N - 1)^2);
    O_C.setLostness(N, S, R, L) ;          // Save lostness for objective first
    Game_N += N ;                     // Summarize for full game lostness value
    Game_S += S;
    Game_R += R;
    Game_L = sqrt((Game_N/Game_S - 1)^2 +
                  (Game_R/Game_N - 1)^2);
    P.reset(C)) ;                      // Start new path from current location
return
```

Algorithm 2 clearly shows the main difference between global and local lostness. Here, in local lostness, it is shown that there is one path for the entire game, rather than a path for each task. Tasks are not considered in this measure, only the objectives. This path, containing unique (N) and total steps (S), is updated each step and, when an objective is found, a breadth-first search (path-finding) is used from the start of the path to the location of the current objective to calculate the minimum number of steps needed to complete it (R). These values are then saved, used to give a value of lostness for the objective, and the path is then reset. Rather than the weighted average used by global lostness, the three lostness variables for each objective, minimum (R), unique (N), and total steps (S), are summed and inputted into the lostness equation to give a lostness value for the full game.

2 SUPPLEMENTARY EXAMPLE

2.1 In-Game Tasks and Objectives

Here, we show how lostness is calculated for a game with three subsequent tasks (see Figure S1):

1. The Chantry (a) – One objective:
   1 Beside Berkeley Castle
2. Gloucestershire (b) – Two objectives:
   1 A County Map
   2 Last Letter to Bristol
3. Smallpox (c) – Three objectives:
   1 Family Grave
   2 Scarred Composer
   3 Monstrous Reflection

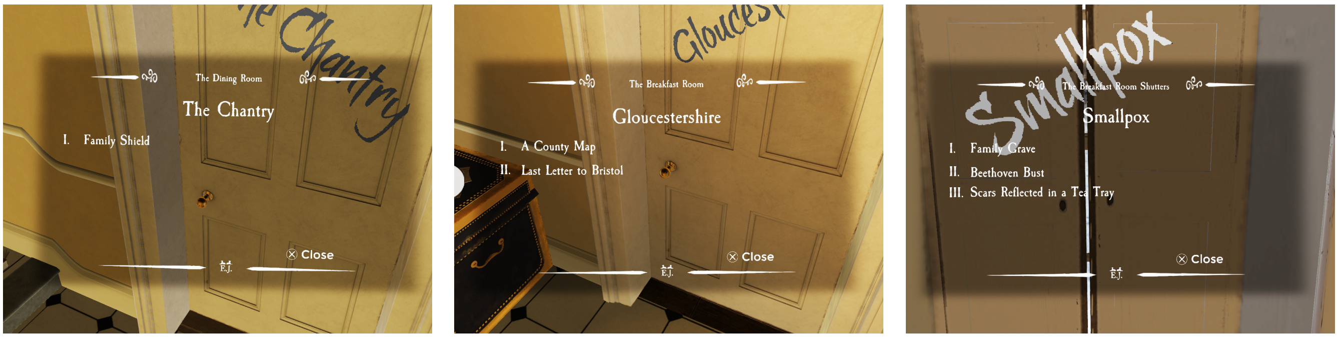

(a) *The Chantry* Task (b) The *Gloucestershire* Task (c) The *Smallpox* Task

Figure S1: Three in-game tasks with their objectives listed, as shown in *the Chantry*.

2.2 Player Steps

In this scenario, the player carries out the following steps:

1. The player goes towards the door to start the Chantry task, they go there directly
Player starts the Chantry task, finds starting objective
(Local Lostness: R = 3, S = 3, N = 3: L = 0)
2. The player briefly goes the wrong direction, taking one extra step, then turns around and goes to the door to start the Gloucestershire task
Player starts the Gloucestershire task, finds starting objective
(Local Lostness: R = 4, S = 5, N = 4: L = 0.2)
3. The player takes an extra step before finding the Last Letter to Bristol objective
Player finds Gloucestershire objective
(Local Lostness: R = 1, S = 2, N = 2: L = 0.5)
4. The player finds the County Map objective and completes the Gloucestershire task
Player finds Gloucester objective (Local lostness: R = 1, S = 1, N = 1: L = 0)
Player completes the Gloucestershire task (Global lostness: R =2, S = 3 , N = 3: L = 0.333)
5. The player takes a long route, going the long way around a table to start the Smallpox task
Player starts the Smallpox task, finds starting objective
(Local lostness: R = 3, S = 6, N = 6: L = 0.5)
6. The player gets completely lost before finding the Family Grave objective
Player finds Smallpox objective
(Local lostness: R = 5, S = 17, N = 12: L = 0.653)
7. The player takes a couple of revisits before finding the Scarred Composer objective
Player finds Smallpox objective
(Local lostness: R = 3, S = 5, N = 3: L = 0.4)
8. The player finds the Monstrous Reflection objective perfectly and completes the Smallpox task
Player finds Smallpox objective (Local lostness: R = 2, S = 2, N =2: L = 0)
Player completes Smallpox task (Global lostness: R = 12, S = 23, N =12: L = 0.506)

9. The player finds the Beside Berkeley Castle objective perfectly and completes the Chantry task
Player finds the Chantry objective (Local lostness: R = 10, S = 10, N = 1: L = 0)
Player completes the Chantry task (Global lostness: R = 3, S = 28, N = 15: L = 0.925)

2.3 Scaling Global Lostness to the Full Game

As detailed in Algorithm 1, the global lostness measure uses a weighted average, using the number of objectives within a task, to scale the lostness value from each task up to the full game. So, according to Equation 2 (see main text), first take the number of objectives and lostness value for each task:

1. The Chantry
    - Number of objectives = 1
    - Lostness = 0.925
2. Gloucestershire
    - Number of objectives = 2
    - Lostness = 0.333
3. Smallpox
    - Number of objectives = 3
    - Lostness = 0.506

$(0.925 \times 1) + (0.333 \times 2) + (0.506 \times 3) = (0.925 + 0.666 + 1.518) = 3.109$

and divide by the sum of objectives $(1 + 2 + 3 = 6)$ to give a value of global lostness (L_G) for the full game:

$L_G = \frac{3.109}{6} = 0.518$

2.4 Scaling Local Lostness to the Full Game

As detailed in Algorithm 2, the local lostness measure sums up the values of R, S, and N values for each objective and feeds these back into the original lostness equation (see Equation 1, main text) to give a value of lostness for the full game. So, according to Equation 3 (see main text), first summarize the R, S, and N values for each objective:

1. Start of the Chantry
    - R: 3
    - S: 3
    - N: 3
2. Start of Gloucestershire
    - R: 4
    - S: 5
    - N: 4
3. Last Letter to Bristol
    - R: 1
    - S: 2
    - N: 2

4. County Map
   - R: 1
   - S: 1
   - N: 1
5. Start of Smallpox
   - R: 3
   - S: 6
   - N: 6
6. Family Grave
   - R: 5
   - S: 17
   - N: 12
7. Scarred Composer
   - R: 3
   - S: 5
   - N: 3
8. Monstrous Reflection
   - R: 2
   - S: 2
   - N: 2
9. Beside Berkeley Castle
   - R: 10
   - S: 10
   - N: 10

So, $R = 32$, $S = 51$, and $N = 43$.

Secondly, input these values into Equation 1 (see the main text) to give a value of local lostness (L_L) for the full game:

$$L_L = \sqrt{\left(\frac{43}{51} - 1\right)^2 + \left(\frac{32}{43} - 1\right)^2} = 0.300, \tag{S1}$$

2.5 Brief Summary

As global lostness has a separate player path for each task, this leads to some noise. This is shown by the high lostness values for the Chantry task due to other tasks being completed before the Beside Berkeley Castle objective was found. The weighted average used for the full game global lostness helps alleviate this but lostness is still higher than the more accurate local lostness measure (0.518 vs 0.300). Moreover, because of the way the player completed the Smallpox task, they ended up revisiting nodes in global lostness, which again contributed to a higher overall lostness. On the other hand, for the Gloucestershire task, there is a perfect global lostness score, despite this not being the case for local lostness. This is

because the extra step taken by the player for the Last Letter to Bristol needed to be taken for the County Map. This issue is addressed more completely in the Discussion section of the main text.
